# Efficacy of a Peruvian Botanical Remedy (Sabell A4+) for Treating Liver Disease and Protecting Gastric Mucosal Integrity

**DOI:** 10.1155/2019/5486728

**Published:** 2019-10-24

**Authors:** Mark G. Swain, John L. Wallace, D. Lorne Tyrrell, José Cabanillas, Steven K. H. Aung, Hongqun Liu, Lindsay Finnie-Carvalho, Grishma Shrestha, Hugh A. Semple, Francis H. Y. Green

**Affiliations:** ^1^Liver Unit, Cumming School of Medicine, University of Calgary, 3280 Hospital Drive N.W, Calgary, Alberta T2N 4Z6, Canada; ^2^Department of Physiology and Pharmacology, Cummings School of Medicine, University of Calgary, 3280 Hospital Drive N.W, Calgary, Alberta T2N 4Z6, Canada; ^3^Li Ka Shing Institute of Virology, Department of Medical Microbiology and Immunology, University of Alberta, Edmonton, Alberta T6G 2J7, Canada; ^4^Sabell Corporation, Suite 1250, 635 8^th^ Avenue SW, Calgary, Alberta T2P 3M3, Canada; ^5^Dr Steven K H Aung Clinic, 9904-106 Street, Edmonton, Alberta T5K 1C4, Canada; ^6^Snyder Institute for Chronic Diseases, Cumming School of Medicine, University of Calgary, 3280 Hospital Drive N.W, Calgary, Alberta T2N 4Z6, Canada; ^7^Alberta Innovates-Technology Futures, Hwy 16A & 75 Street, Vegreville, Alberta T9C 1T4, Canada; ^8^Department of Pathology and Laboratory Medicine, Cumming School of Medicine, University of Calgary, 3280 Hospital Drive N.W, Calgary, Alberta T2N 4Z6, Canada

## Abstract

The purpose of this study was to determine the efficacy of a Peruvian botanical formulation for treating disorders of hepatic function and gastric mucosal integrity. The formulation A4+ (Sabell Corporation) contains extracts of *Curcuma longa* rhizome, *Cordia lutea* flower, and *Annona muricata* leaf. Individually these plants have been used as traditional remedies for liver disease. We report the efficacy of A4+ and its components using a variety of in vitro and in vivo disease models. The methods used included tests for antioxidant, anti-inflammatory, and antiviral activity as well as mouse models of liver disease, including Concanavalin A-induced immune-mediated hepatitis and a bile duct ligation model for evaluating sickness behaviour associated with liver disease. Rat models were used to evaluate the gastric mucosal protective property of A4+ following indomethacin challenge and to evaluate its anti-inflammatory action in an “air pouch” model. In all tests, A4+ proved to be more effective than placebo. A4+ was antioxidant and anti-inflammatory and diminished Hepatitis C virus replication in vitro. In animal models, A4+ was shown to protect the liver from immune-mediated hepatitis, improve behavioural function in animals with late stage liver disease, and protect the rat gastric mucosa from ulceration following NSAID exposure. We conclude that A4+ ameliorated many aspects of liver injury, inhibited hepatitis C virus replication, and protected the gastric mucosa from NSAIDs. These varied beneficial properties appear to result from positive interactions between the three constituent herbs.

## 1. Introduction

A4+ is an herbal product formulated to support healthy liver function. It contains extracts of three herbs: *Curcuma longa* rhizome, *Cordia lutea* flower, and *Annona muricata* leaf, combined in the *w*/*w* ratio of 10 : 80 : 10. This unique combination of herbs and their relative proportions was developed by Dr. José Cabanillas, a Peruvian physician with extensive experience of traditional medicine in the Amazon basin. The herbal components originate from the Amazon rainforest and the Coastal plains of Peru. Individually these plants have been used as a source of traditional remedies in many countries, and monographs have been published supporting the use of *Curcuma longa* and *Annona muricata* for the treatment of patients with liver disorders [1, 2]. *Cordia lutea* has also been used traditionally to treat hepatitis [3]. A4+ is licensed by the Natural Health Product Directorate of Health Canada (licence NPN 880033347) to support healthy liver function [4]. We have previously reported on the toxicology and chemistry of A4+ [5].

Curcumin is a polyphenolic compound present in *Curcumin longa* with known anti-inflammatory, antiviral, antioxidant, and anticancer activities [6]. It is a well-studied phytochemical, largely due to its excellent safety profile and its wide range of potential applications. *Annona muricata*, another constituent of A4+ formulation, has also been well studied, with its extracts showing hepatoprotective and chemotherapeutic potential [7]. In contrast, pharmacodynamic and pharmacokinetic properties of *Cordea lutea* are not well characterized.

The purpose of the studies reported here was to determine the effectiveness of A4+ in treating a variety of experimental liver diseases and gastric ulceration resulting from nonsteroidal anti-inflammatory drugs (NSAIDs) and determine its activity against hepatitis B and C viruses using in vitro and in vivo models. We report that A4+ shows protective effects against gastric ulceration, immune-mediated hepatitis, and systemic behavioural effects of liver disease. We also report that A4+ has potent antiviral activity against hepatitis C but not hepatitis B virus.

## 2. Methods/Design

A4+ was extracted from the individual plants as previously described [5]. In vitro and animal studies were conducted to determine the potential for A4+ to ameliorate liver disease and one study to assess its ability to protect the gastric mucosa from NSAIDs. The doses selected for these assays were largely based on the individual investigators' experience with the individual assays but also to be within the range of safety described in the toxicology studies [5]. The recommended human adult daily dose of A4+ (533 mg/day) as a Natural Health Product was also taken into account [4].

All procedures in these studies were approved by the Animal Care Committees of the University of Calgary and University of Alberta and were performed in accordance with the guidelines established by the Canadian Council on Animal Care.

### 2.1. Evaluation of the Hepatoprotective and Behavioural Effects of the Herbal Compound A4+ in Two Models of Liver Injury

#### 2.1.1. Behavioural Studies for Evaluating Sickness Behavior

The bile duct ligation model in mice is an established method for evaluating sickness behaviour associated with liver disease [8]. Male C57BL/6 mice were randomly divided into two groups: bile duct resection (BDR) surgery only (control) and BDR plus A4+ (160 mg/kg/day administered by oral gavage for 9 days). Behavior studies were conducted one day before surgery and 5 and 9 days after the operation. Sickness behavior was evaluated using two well-established methods. A social investigation paradigm was used to examine the loss of social interest [9] and open field locomotor activity measurement to assess overall mobility [10]. Student's *T* test was used for statistical analysis, and a *p* value of less than 0.05 was considered significant. Additional details are provided in Supplemental Methods [Supplementary-material supplementary-material-1].

#### 2.1.2. Concanavalin A- (Con A-) Induced Hepatitis

The second liver injury model was Concanavalin A- (Con A-) induced immune-mediated hepatitis [11, 12]. One group of male C57BL/6 mice were pretreated with saline vehicle (control) and another group with A4+ (640 mg/kg) via oral gavage. The groups received their respective treatments 3 days, 2 days, 1 day, and one hour before being given Con A (13.5 mg/kg) intravenously. Twelve hours after injection with Con A, mice were euthanized and blood was collected for measurement of plasma alanine transaminase (ALT), as an index of acute liver injury [11, 12]. Other markers of liver injury were not studied. Additionally, livers were dissected and processed for flow cytometry analysis (FACS) after staining of different cell surface markers to identify cytokine production profiles of these cells using intracellular staining. Student's *T* test was used for statistical analysis, and a *p* value of less than 0.05 was considered significant. Additional details are provided in Supplemental Methods [Supplementary-material supplementary-material-1].

### 2.2. Evaluation of the Antioxidant, Anti-inflammatory, and Mucosal Protective Actions of A4+ and Its Constituents

#### 2.2.1. Antioxidant Activity

The antioxidant activity of A4+ and its constituents was evaluated using an in vitro assay in which a stable free radical (DPPH) was allowed to interact with the test substance [13]. In the presence of free radical scavengers (i.e., antioxidants), there is a detectable decrease in absorbance. The compounds tested were *Cordia*, *Annona*, and *Curcuma* extracts. Changes in absorbance at 540 nm were recorded every minute over a 10-minute period. Ten separate experiments were carried out, and the results are reported as mean ± SEM. Groups of data were compared using ANOVA followed by Dunnett's multiple comparison test. A *p* value of less than 0.05 was considered significant. Additional details of the methodologies used in the experiments above are available in the online Supplemental Methods Sections [Supplementary-material supplementary-material-1].

#### 2.2.2. Anti-Inflammatory Activity

The anti-inflammatory action of A4+ was evaluated using a widely used in vivo “air pouch” model [14]. A subdermal “pouch” was created on the back of the rat by repeated injection of air [14]. After the pouch had been created, an inflammatory event was triggered by injecting zymosan. The test compounds were administered either directly into the air pouch or orally 2 hours prior to injection of zymosan. The rats (*n* = 5 or 6) were euthanized 4 h after injection of zymosan, and the exudates were withdrawn from the air pouch for analysis by ELISA [14]. The number of leukocytes in the exudates was determined using a Sysmex KX-21N Hematology analyzer. In addition to a negative control (vehicle), indomethacin was included as a positive control in these experiments. Groups of data were compared using ANOVA followed by Dunnett's multiple comparison test. A *p* value of less than 0.05 was considered significant. Additional details of the methodologies used in the experiments above are available in the online Supplemental Methods Sections [Supplementary-material supplementary-material-1].

#### 2.2.3. Mucosal Protective Activity

The potential mucosal protective effects of A4+ and its constituents were assessed at three concentration levels. For this study, groups of 5 male Wister rats were used and they were deprived of food, but not water, for 18–20 h prior to oral administration of indomethacin (20 mg/kg). They were treated orally with A4+ at doses of 50, 300, or 1000 mg/kg 4 hours prior to oral indomethacin administration. The rats were sacrificed, and the extent of haemorrhagic damage in the stomach was assessed in a blind manner 3 hours after indomethacin administration. This involved measuring the lengths of all lesions in mm and then summing those lengths to give a “gastric damage score” for each rat [15, 16]. Groups of data were compared using ANOVA followed by Dunnett's multiple comparison test. A *p* value of less than 0.05 was considered significant.

### 2.3. Antiviral Effects of A4+ Plant Extracts on HCV- and HCB-Infected Cells

#### 2.3.1. Hepatitis C Studies

The studies were carried out using Huh7.5 cells and a tissue-culture adapted strain of HCV and JFH. Cells were seeded and infected with the HCV strain followed by treatment with A4+ (0.1, 0.5, 1, 5, and 10 *μ*g/mL in 45% ethanol) for 4 days. Two control groups were established: ethanol and Negative. Ethanol without A4+ diluted in it was used for the ethanol group, and fresh cell medium was used for the negative control group. The studies were repeated three times, and intracellular and extracellular fractions were measured 4 days after the herbal treatment.

Additionally, viral protein levels were visualized by western blot to further assess the antiviral effects of A4 + L. Cells were plated and infected, and herbal treatment was conducted as previously described. After treatment, cells were lysed with RIPA buffer to release cell contents and prevent protein degradation. Protein levels were quantified with a BioRad Protein assay, and NS3 and core HCV antibodies were used to determine viral quantity. ANOVA followed by Dunnett's multiple comparison test was used for statistical analysis, and a *p* value of less than 0.05 was considered significant. Additional details of the methodologies used in the experiments above are available in the online Supplemental Methods [Supplementary-material supplementary-material-1].

#### 2.3.2. Hepatitis B Studies

The HBV antiviral studies used both HepG2.2.15 and HepAD38 cells, which express HBV constitutively under the presence of specific promoters. ANOVA followed by Dunnett's multiple comparison test was used for statistical analysis, and a *p* value of less than 0.05 was considered significant. Additional details of the methodologies used in the experiments above are available in the online Supplemental Method [Supplementary-material supplementary-material-1].

#### 2.3.3. Viability Assays

Cell viability during herbal treatment was determined using an MTT assay. As the number of cells increase, the amount of MTT metabolized also increases, resulting in a larger quantity of purple formazan formation, and therefore the development of a higher absorbance read at 570 nm. ANOVA followed by Dunnett's multiple comparison test was used for statistical analysis, and a *p* value of less than 0.05 was considered significant. Additional details of the methodologies used in the experiments above are available in the online Supplemental Method [Supplementary-material supplementary-material-1].

#### 2.3.4. Effect of Short-Term Exposure to A4+ Plant Extract on Natural Killer Cell Activity

This study evaluated whether potential antiviral effects of the A4+ plant extract might be due to enhancement of Natural Killer (NK) activity. C57B/6 mice were given 100 *μ*L of sucrose, ethanol, or A4+ (1.3 mg/day) by gavage daily for 14 days. After 14 days, mice were euthanized and their spleens removed for NK cell preparation. A cell suspension was prepared, and NK cells were purified using an Easy Sep Mouse NK Cell Enrichment kit. Purified NK cells were analyzed by FACS [17]. Student's *T* test was used for statistical analysis, and a *p* value of less than 0.05 was considered significant. Additional details of the methodologies used in the experiments and an example of FACS analysis are available in the online Supplemental Method [Supplementary-material supplementary-material-1] and online Supplemental [Supplementary-material supplementary-material-1].

## 3. Results

### 3.1. Evaluation of the Hepatoprotective and Behavioural Effects of the Herbal Compound A4+ in Two Liver Injury Models

#### 3.1.1. Rationale

Patients with liver disease often exhibit a number of associated symptoms including fatigue, malaise, loss of interest in engaging in social activity, and inability to concentrate, which have been collectively termed sickness behaviors [18, 19]. The purpose of these studies was to investigate whether A4+ could attenuate the sickness behavioural and biochemical effects associated with liver injury in two well-characterized mouse models of liver injury.

#### 3.1.2. Results

**Figure 1:**
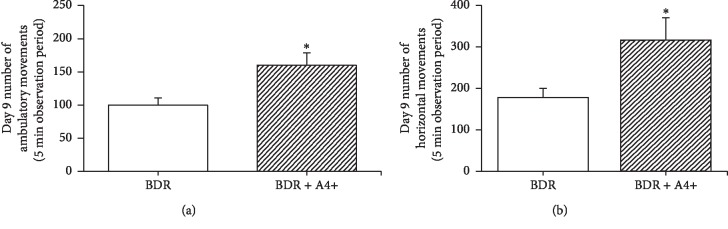
(a) Number of ambulatory movements exhibited by day 9 BDR + vehicle (BDR) *vs* day 9 BDR + A4+ (160 mg/kg/day) treated mice after being placed in an open field apparatus. Observation period was 5 minutes. Bars are the mean ± SD of data from 7 BDR + vehicle and 8 BDR + A4^+^ treated mice per group. ^*∗*^*p*=0.03 vs BDR + vehicle group. (b) Number of horizontal movements exhibited by day 9 BDR + vehicle (BDR) *vs* day 9 BDR + A4+ (160 mg/kg/day) treated mice after being placed in an open field apparatus. Observation period was 5 minutes. Bars are the mean ± SD of data from 7 BDR + vehicle and 8 BDR + A4^+^ treated mice per group. ^*∗*^*p*=0.04 vs BDR + vehicle group.

The first bile duct ligation and resection (BDR) model in mice showed that plasma ALT and total bilirubin levels were similar in control and A4+ treated BDR mice at 9 days after surgery, as was time spent in social investigation behaviour (Supplemental [Supplementary-material supplementary-material-1]). In contrast, BDR animals administered A4+ for 9 days were significantly more active in overall mobility for both ambulatory movements (*p*=0.03) and enhancement of number of horizontal movements (*p*=0.04) (Figures 1(a) and 1(b)).

### 3.2. Evaluation of the Immune Hepatoprotective Effect of Herbal Compound A4+ in Concanavalin a- (Con A-) Induced Hepatitis

#### 3.2.1. Rationale

Concanavalin A-induced hepatitis is a well-characterized and widely used model of T cell-mediated hepatitis mimicking many aspects of human T cell-mediated liver disease, including autoimmune hepatitis and viral hepatitis [11, 12]. The effects of A4+ on the hepatotoxic and immune-mediated effects of Concanavalin A were studied in a mouse model. The hepatitis is characterized by hepatocellular necrosis and inflammatory cell infiltration with marked increase in plasma ALT 8 hours after Con A treatment [11].

#### 3.2.2. Results

**Figure 2:**
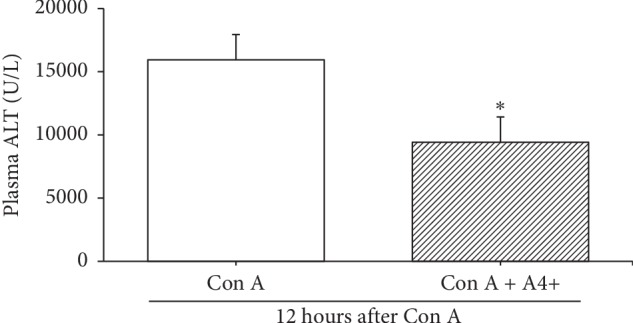
Plasma ALT levels were measured 12 hrs after Con A (13.5 mg/kg iv) treatment in mice which received either saline vehicle (Con A) or A4+ (640 mg/kg) + Con A by oral gavage 1 hr prior to Con A treatment. Bars are the means ± SD of data from 7 Con A plus vehicle mice, and 9 Con A plus A4+ mice, per group. ^*∗*^*p*=0.04 vs Con A plus vehicle group.

A4+ treated Con A mice showed significant declines in plasma ALT (*p*=0.04) compared to Con A (alone) treated mice (Figure 2).

**Figure 3:**
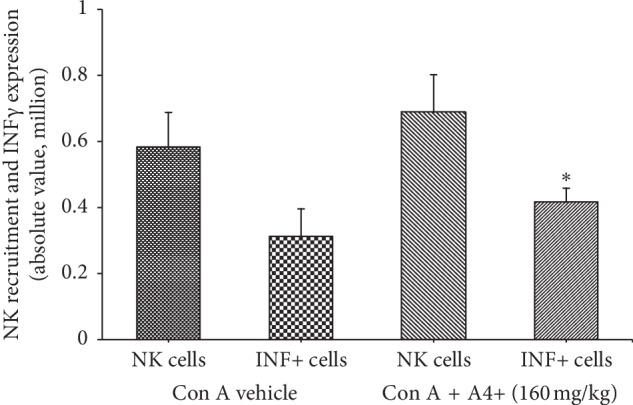
Hepatic NK cell (determined as NK1.1^+^ CD3^−^ cells by FACS) recruitment to the liver and activation (i.e., IFN*γ* expression by FACS). Bars are the mean ± SD of data from *n* = 6 mice per group. Total NK cell recruitment (in millions of cells per liver) was similar in Con A plus vehicle-treated vs Con A plus A4+ treated groups. NK cells expressing IFN*γ* were significantly increased in Con A plus A4+ treated mice compared to Con A plus vehicle-treated mice; ^*∗*^*p* < 0.05.

Pretreatment with A4+ had no significant effect on total hepatic IFN cell recruitment (Figure 3). In contrast, hepatic recruitment of a subpopulation of NK cells expressing IFN*γ* were significantly (*p* < 0.05) increased in the A4+ Con A treated animals compared to Con A controls (Figure 3).

### 3.3. Evaluation of the Antioxidant, Anti-Inflammatory, and Mucosal Protective Actions of A4+ and Its Constituents

#### 3.3.1. Rationale

In this section, we explored the antioxidant, anti-inflammatory, and mucosal protective effects of A4+. These properties are likely to be beneficial in treating liver and mucosal diseases.

#### 3.3.2. Results

**Figure 4:**
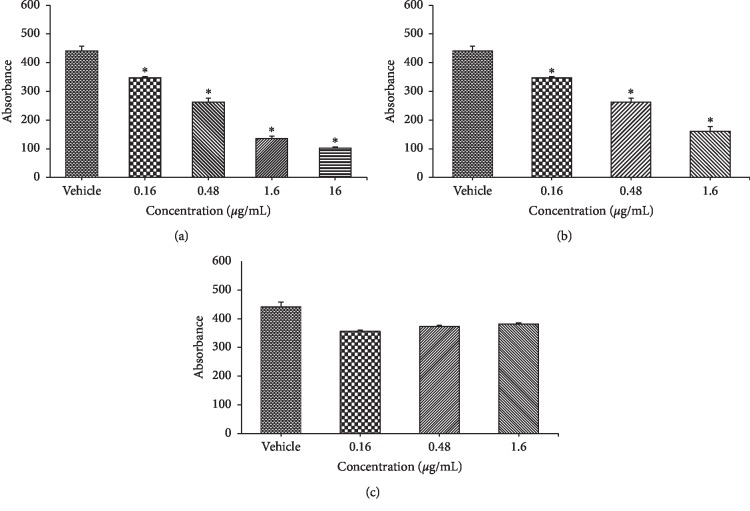
In vitro antioxidant activity of constituents of A4+. Each bar shows the mean ± SEM of 10 separate experiments. The antioxidant activity of A4+ was mainly attributable to the *Cordia* and *Annona*. Note that *Curcuma* and *Annona* did not remain in solution at concentrations >1.6 *μ*g/ml. *∗*Statistically significant from the vehicle. (a) *Cordia*. (b) *Annona*. (c) *Curcuma*.

*(1) Antioxidant Activity*. An in vitro assay was used to evaluate the antioxidant activity of A4+ and its constituents [13]. Antioxidant activity was exhibited by *Cordia* and *Annona*, which showed significant dose response relationships (*p* < 0.01). There was no significant contribution from the *Curcuma* extract (Figure 4).

**Figure 5:**
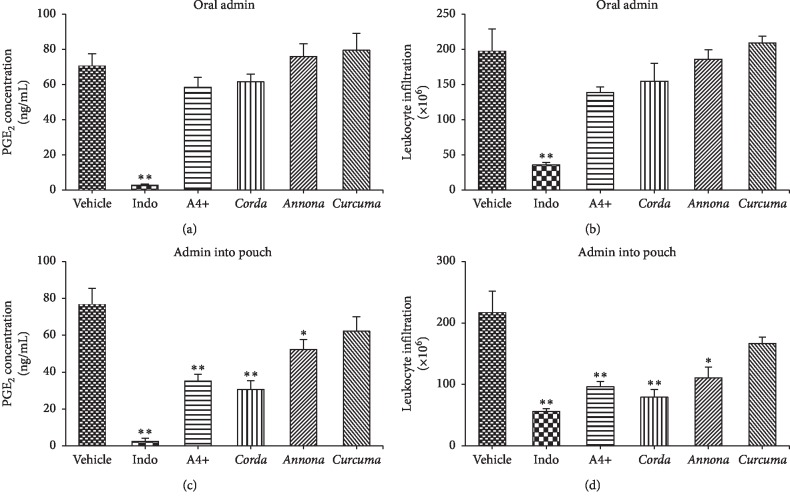
Effects of A4+ and its constituents on zymosan-induced PGE_2_ concentration with (a) oral or (b) pouch administration, and leukocyte infiltration with (c) oral or (d) pouch administration in the rat air pouch model. Results are shown as mean ± SEM (*n* = 5-6 rats/group). ^*∗*^*p* < 0.05, ^*∗∗*^*p* < 0.01 versus the vehicle-treated group (ANOVA and Dunnett's multiple comparison test).

*(2) Anti-Inflammatory Activity*. The antiinflammatory action of A4+ was evaluated using an in vivo “air pouch” model [14]. This model determines the effect of a drug on many different aspects of the inflammatory process. Administration of A4+ directly into the air pouch at a dose of 10 mg/kg markedly reduced PGE_2_ (Figure 5(b)) and leukocyte infiltration (Figure 5(d)) (*p* < 0.01). This effect was similar to that of the nonsteroidal anti-inflammatory drug indomethacin at 1 mg/kg. The *Corda lutea* (8 mg/kg) and *Annona muricata* (1 mg/kg) constituents of A4+ produced effects similar to A4+, but no significant effect was observed with *Curcuma longa* (1 mg/kg). Oral administration of A4+ did not result in significant reductions of the inflammatory mediators, PGE_2_ (Figure 5(a)), and leukotriene B_4_ (Supplemental [Supplementary-material supplementary-material-1]), or acute inflammatory cells (Figure 5(c)) into the air pouch.

**Figure 6:**
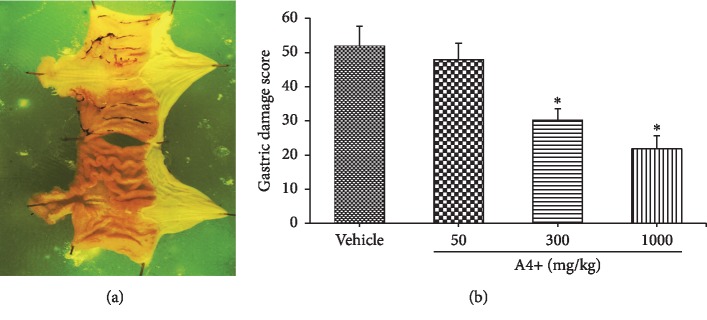
(a) Hemorrhagic stomach mucosa following indomethacin administration, in a positive control animal. (b) Mucosal ulceration score of rats treated orally with A4+ at doses of 50, 300, or 1000 mg/kg 4 hours prior to indomethacin. There were significant reductions in hemorrhage at doses above 300 mg/kg. ^*∗*^*p* < 0.05 versus the vehicle-treated group.

*(3) Mucosal Protective Activity*. Ulceration in the gastrointestinal tract is a significant clinical problem. It is a major limitation to the use of nonsteroidal anti-inflammatory drugs (NSAIDs) for the treatment of disorders such as arthritis. Thus, there is substantial clinical need for agents that can reduce the ulcerogenic effects of NSAIDs. The mucosal-protective effects of A4+ and its constituents were examined by assessing the extent of attenuation of haemorrhagic damage in the stomach caused by indomethacin [15], as shown in Figure 6(a). Oral A4+ administration reduced the severity of gastric damage induced by administration of the potent nonsteroidal anti-inflammatory drug (indomethacin) in dose-dependent manner, and this effect was only significant at doses of ≥300 mg/kg, when given 4 hours prior to the indomethacin (Figure 6(b) and Supplemental [Supplementary-material supplementary-material-1]).

### 3.4. Antiviral Effects of A4+ Plant Extracts on HCV- and HCB-Infected Cells

#### 3.4.1. Hepatitis C Studies

*(1) Rationale*. Hepatitis C (HCV) is a viral infection of the liver, which can cause chronic hepatitis and hepatocellular carcinoma. The virus contains a single-stranded positive-sense RNA genome that encodes 10 structural or nonstructural proteins. Many of these proteins have the capacity to interfere with cell signaling during infection and can effectively take over the cell for viral replication [20].

The purpose of the first part of this study was to examine for an antiviral effect of A4+ on hepatitis C virus- (HCV-) infected Huh 7.5 cells.

**Figure 7:**
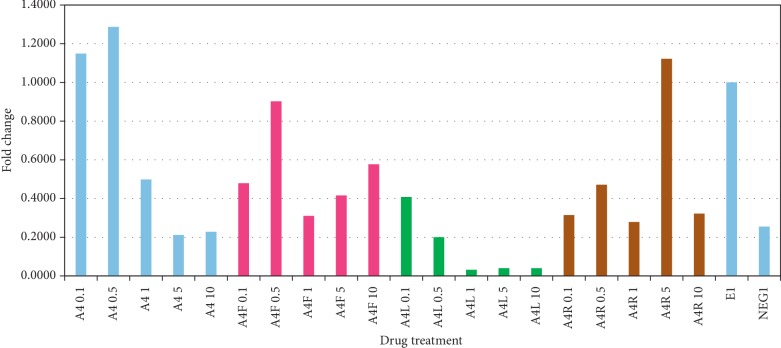
Effect of A4+ and its constituents on intracellular HCV titers. Huh 7.5 cells were exposed to diluted A4+ powder or its constituents (0.1, 0.5, 1, 5, and 10 *μ*g/mL in 45% ethanol) for 4 days. A4: A4+; A4F: *Cordia*; A4L: *Annona*; A4R: *Curcuma*; E: ethanol control; NEG: cell media only. This is a representative plot of one of three separate experiments. A4+ shows a dose-related reduction in HCV titers (blue bars on the left). This effect was attributed to the *Annona* component (green bars).

*(2) Results*. A4+ powder and in particular the A4 + L component of the formulation had significant (*p* < 0.05) antiviral activity. Intracellular and extracellular fractions were measured after 4 days of herbal treatment, and both fractions showed a significant drop of HCV titers at A4 + L concentrations greater than 1 *μ*g/mL. A4 + L showed antiviral activity at all concentrations examined but was most active at 1 *μ*g/ml or greater, with approximately 90% inhibition of HCV in cell cultures at 10 *μ*g/ml (Figure 7).

**Figure 8:**
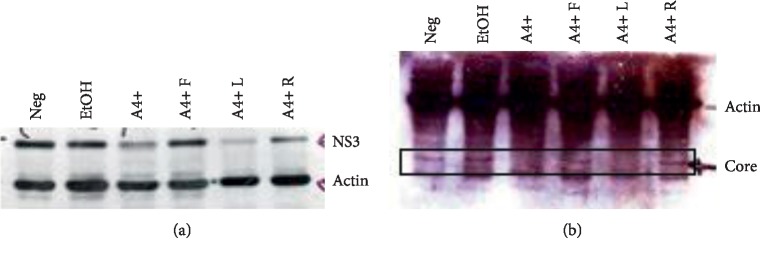
Western blots of 7.5 cell lysates infected with TCA-HCV followed by a 4-day drug treatment at a concentration of 10 *μ*g/mL. (a) was run on a 10% polyacrilamide gel, and (b) was run on a 15% polyacrilamide gel. This is a representative image of one of three separate experiments.

To further assess the antiviral effects of A4 + L, viral protein levels were visualized by western blot. A drop in NS3 is seen in lanes for A4+, A4 + L, and A4 + R, with the greatest reduction observed for A4 + L (Figure 8). This observation indicates that viral load was decreased as a result of A4+ exposure.

#### 3.4.2. Hepatitis B Studies

Only A4 + L was tested in these assays, and no antiviral effect on HBV was demonstrated.

#### 3.4.3. Viability Assays

All herbs were tested on infected cells to determine if they restricted cell growth. Although not a toxicity study, this can be a valuable tool to discover if a drug has a negative impact on cell proliferation. As the number of cells increase, the amount of MTT metabolized also increases, resulting in a larger quantity of purple formazan and a higher absorbance. The herbal treatment is quite short, only allowing for an immediate effect on the cells to be seen. Most of the herbs tested showed no cell toxicity. The tincture of A4 + L decreased the absorbency by approximately 1/3 at a dilution of 10^−7^. However, this effect was not seen when the powder of A4 + L was tested, although the initial preparation was similar. The difference in the two lots of A4 + L toxicity was likely related to the presence of ethanol used in the tincture.

#### 3.4.4. Effect of Short-Term Exposure to A4+ Plant Extract on Natural Killer Cell Activity

*(1) Rationale*. The purpose of this study was to determine if potential antiviral effects of the A4+ plant extract might be attributed to enhancement of natural killer activity.

*(2) Results*. Treatment with A4+ did not enhance NK activity in vivo (Supplemental [Supplementary-material supplementary-material-1]). Therefore, since the antiviral activity of A4+ was only shown in a lymphocyte-free cell culture system, it was concluded that the antiviral effect of A4+ on HCV is a direct antiviral effect and is not mediated through a NK immune enhancement. Additional details of the results used in the above experiments, including statistical tests, are available in the online Supplemental [Supplementary-material supplementary-material-1] and Supplemental [Supplementary-material supplementary-material-1].

No toxicity attributable to A4+ or its constituents was observed in any of the animal or in vitro assays reported above.

## 4. Discussion

Herbal remedies have been used traditionally worldwide for treating and preventing liver disorders. The most studied herbs in this area are *Silybum marianum* (milk thistle) seed, *Glycyrrhiza glabra* (licorice) root, *Cinnamomum zeylanicum* (cinnamon) bark, and *Phyllantus* spp. among many others [21, 22]. The range of applications of these herbs for hepatic disorders is wide, but they are predominantly used to improve well-being and quality of life and help manage side effects related to antiviral treatments [22]. Additionally, there are numerous preclinical and clinical studies that suggest these herbal remedies could help patients with nonalcoholic fatty liver disease (NAFLD), viral hepatitis, alcohol liver disease, and drug induced hepatotoxicity [21, 22].

In this study, using a variety of in vitro and animal models, we show that A4+ ameliorates many aspects of liver injury through its behavioural, anti-inflammatory, and antiviral effects. In addition, we show that A4+ has potential for preventing gastric mucosal hemorrhage following the ingestion of NSAIDs. We attribute this broad range of effects to the composition of the formulation which includes three herbs, *Curcuma longa* rhizome, *Cordia lutea* flower, and *Annona muricata* leaf, all of which have been used traditionally for treating liver disease [1–3].

The in vitro studies showed that the individual herbs had some distinct properties, thus many of the benefits of the formulation may result from the combined effects of the three constituent herbs. For example, we show that the antiviral effect of A4+ is largely due to the *Annona muricata*, the anti-inflammatory effect is largely due to *Cordia lutea* flower and *Annona muricata* leaf, and the anti-oxidant activity was demonstrated by the *Annona muricata* and *Cordia lutea* species. Studies of the individual herbs were not performed in all of the animal experiments in order to reduce the number of animals sacrificed. The differences of the effects of individual herbal constituents in these assays are probably best explained by differences in their chemical composition [5].

The specific effects of *Cordea lutea* are the least well researched of the three herb components. Several studies have shown the hepatoprotective potential of other species of the *Cordia* family [3, 23, 24]. The flower component of *Cordia lutea* has been used as a traditional medicine for centuries in Peru [1]. Our previous study indicates that one of the compounds contained in *Cordea lutea* flower extract is similar to rutin, with an additional 30 components unidentified [5]. The extract of *Annona muricata* leaf was also found to contain a rutin-like compound [5]. Rutin is a well-studied flavonoid with diverse therapeutic properties including anti-inflammatory, antiviral, antibacterial, antiulcer, and hepatoprotective effects [25, 26]. The antiulcer activity of rutin is attributed to a concentration-dependent inhibition of a gastric proton pump [27]. In animal studies, the gastroprotective effects of rutin in both indomethacin and ethanol-induced gastric mucosal damage were associated with inhibition of neutrophil infiltration and enhanced antioxidant activity [28, 29]. Thus, the potent gastric mucosal protective activity of A4+ could, at least in part, be due to rutin or rutin-like compounds present in the plant extracts of the *Cordia lutea* and *Annona muricata* constituents of A4+.

The seed, fruit, leaves, and bark of *Annona muricata* have been used traditionally for treating numerous ailments from heart and liver disorders to malaria [7]. The leaves and the seeds are well studied as they have significance in traditional use. Studies have identified 212 bioactive compounds in *A. muricata* with acetogenins, alkaloids, and phenols being predominant [7]. Antiviral activities of this plant extract against Herpes Simplex I and HIV-I virus have been reported and hypothesized to be mediated by polyphenol compounds [7]. In our in vitro studies, *A. muricata* leaf extract was found to have potent antiviral activity against HCV, but without a demonstrable antiviral effect against HBV. The specificity of this antiviral effect remains unexplained.

Hepatoprotective activity of *A. muricata* extract has been compared to that of silymarin (a component of milk thistle) and is effective against hyperbilirubinemia and acetaminophen-induced hepatotoxicity [7]. *A. muricata* also contains quercetin, another flavonoid [5]. Quercetin is a potent antioxidant with anti-inflammatory and with antiviral activity [30]. An antioxidant-mediated antiulcer activity of *A. muricata* extract has also been reported [7].

Curcumin has shown gastroprotective activity in animal models of indomethacin-induced gastric ulceration [31]. This activity may be attributed to curcumin's ability to enhance the gastric mucosal barrier and reduce gastric acid secretion [31]. A major challenge of using *Curcuma longa* is the low oral bioavailability of curcumin. Pharmacokinetic studies attribute this quality to several factors including low solubility, poor intestinal permeability, and extensive first-pass intestinal and hepatic metabolism [32]. Preclinical and clinical studies of curcumin have demonstrated coadministration of piperine (present in the fruit of the pepper vine (*Piper nigrum*)) substantially increases its bioavailability [33]. In humans, Shoba et al. [34] found an increase in curcumin bioavailability by 2000% when 20 mg of piperine was coingested with 2 g of pure curcumin powder, without any adverse effects. This increase in bioavailability has been attributed to piperine's inhibition of hepatic and intestinal glucuronidation, a pathway extensively involved in curcumin metabolism [33, 34]. Future studies of A4+ coadministered with piperine might show improvements in efficacy.

Although pharmacodynamic and pharmacokinetic interactions were not specifically examined in this study, there is a wealth of evidence that whole plant extracts have different properties than can be predicted from the individual components. A good example of the emergent properties of whole plant extracts was demonstrated by Yang et al. [35]. The investigators studied the effects of whole leaf extract from *Annona muricata* in in vivo and in vitro models of prostate cancer. They found whole leaf extract was more effective in inhibiting cancer cell proliferation compared to a flavonoid enriched extract of *Annona muricata*. Furthermore, an acetogenin-enriched extract exhibited severe toxicity compared to the whole leaf extract. Another study of particular relevance to our study used five individual herbs including *Curcuma* in a gastric mucosal protection model similar to the one described in this paper [36]. The investigators showed that the combination of herbs exhibited synergistic gastric mucosal protection compared to the individual herbs at equivalent doses.

A key issue with many herbal preparations is their potential for hepatotoxicity, and several dozen herbs have been implicated in causing liver injury [37]. An approximately equal number of herbs have been shown to have beneficial effects on liver disease [38]. We have previously shown that A4+ has an excellent safety profile with no demonstrable toxic effects, even in a 28-day repeated dose study ranging up to 2,000 mg/kg orally, the maximum permissible level for regulatory purposes [5]. A no-observed-adverse-effect level (NOAEL) of 2,000 mg/kg was assigned to A4+ [5]. Although safety was not the primary objective of the studies reported here, we found no evidence of toxicity in any of the in vitro or animal studies.

As mentioned earlier, patients with liver disease often exhibit a number of associated symptoms [9] which have been collectively termed sickness behaviors [18, 19]. The studies reported here indicate that A4+ had beneficial effects in tests which examined liver disease-associated sickness behaviours [10]. These improvements occurred in the absence of significant changes in biochemical indices of liver damage in the BDR model.

The immune-mediated hepatitis studies showed that A4+ treatment significantly attenuated Con A hepatitis as reflected by a reduction in plasma ALT levels compared to vehicle-treated controls. In addition, significantly more hepatic NK cells expressed IFNγ in mice pretreated with A4+ which received Con A than in vehicle-treated mice. IFN*γ* has antiviral effects [39]. We also showed a direct antiviral effect of A4+ on HCV-infected cells in vitro, an unexpected finding that was attributable to the *Annona muricata*.

Chronic liver diseases are a major worldwide problem and take many forms, most of which lack effective treatment [38]. An example of this is nonalcoholic fatty liver disease (NAFLD), estimated to affect approximately 6–35% of the adult world population, a proportion of whom will progress to steatohepatitis, cirrhosis and liver cancer [38, 40]. Several studies indicate that herbal remedies can be beneficial in preventing progression of the disease [38]. A recent study of curcumin in a randomized double-blind placebo controlled trial showed that curcumin was associated with a significant reduction in liver fat content (assessed through ultrasonography) [40]. They showed a 78.9% improvement in the curcumin group versus 27.5% improvement in the placebo group [40]. There were also significant beneficial changes in body mass index and liver enzymes [40]. One of the components of A4+ is a Peruvian variety of *Curcumin longa* which contains curcumin [5]. A4+ may prove to be beneficial for patients with NAFLD, clinical trials are indicated.

## 5. Conclusions

In summary, we show that A4+ and its constituent herbs exhibit a variety of potentially beneficial effects on hepatic diseases and gastric mucosal integrity. Specifically, A4+ showed potent antioxidant and anti-inflammatory activity, was protective against immune-mediated hepatitis, and improved sickness behaviour in a model of chronic liver disease. A4+ demonstrated potent antiviral activity against hepatitis C. This effect was shown to be due to the *Annona muricata* leaf. Finally, A4+ proved highly effective at protecting rat gastric mucosa from the ulcerogenic effects of NSAIDs. The individual herbs had differing effects in the in vitro assays indicating that the range of beneficial effects resulted from this unique combination of herbs.

## Data Availability

The data used to support the findings of this study are available from the corresponding author upon request.

## Abbreviations

A4F: *Cordia lutea* flower
A4L: *Annona muricata* leaf
A4R: *Curcuma longa* root
ALT: Alanine aminotransferase
ANOVA: Analysis of variance
BDR: Bile duct ligation and resection
Con A: Concanavalin A
DPPH: 2,2-diphenyl-1-picrylhydrazyl
ELISA: Enzyme-linked immunosorbent assay
FACS: Fluorescence-activated cell sorter
HBV: Hepatitis B virus
HCV: Hepatitis C virus
IFN: Interferon
IFN*γ*: Interferon gamma
MTT: 3-(4,5-dimethylthiazol-2-yl)-2,5-diphenyltetrazolium bromide
NK: Natural killer
NAFLD: Nonalcoholic fatty liver disease
NOAEL: No observed adverse effect level
NS3: Nonstructural protein 3
NSAIDs: Nonsteroidal anti-inflammatory drugs
PGE_2_: Prostaglandin E_2_
RIPA: Radioimmunoprecipitation
SD: Standard deviation.

## Supplementary Materials

Supplementary Materials The supplemental file includes methods and results sections. The methods section provides more detailed descriptions of methods used for the experiments discussed in the manuscript. Furthermore, it provides details on additional experiments conducted that were relevant but not central to our studies. The results section in the supplementary file includes figures and tables that are referenced in the manuscript, which are relevant but were not the primary focus of the research. Specifically, the methods used to examine the loss of social interest in the bile duct ligation mouse model of liver disease and the results following A4+ administration are presented (Supplemental methods section 1 and Supplemental results Figure 1). Effects of A4+ and its constituents on zymosan-induced leukotriene B4 production in the rat air-pouch model are also presented (Supplemental results Figure 1). As flow cytometry was used to study the effect of A4+ plant extract on natural killer cell activity, an example of fluorescence-activated cell sorting (FACS) image of a negative control sample is included in Supplemental Figure 4. The effect of A4+ plant extract on natural killer cell activity is presented in Supplemental Table 1.
