# Supplementary material for: Efficacy of a Peruvian Botanical Remedy (Sabell A4+) for Treating Liver Disease and Protecting Gastric Mucosal Integrity
Source: Evid Based Complement Alternat Med. 2019 Oct 24;2019:5486728. doi: 10.1155/2019/5486728 (PMC6855027; doi:10.1155/2019/5486728)
Supplement: Supplementary Materials — The supplemental file includes methods and results sections. The methods section provides more detailed descriptions of methods used for the experiments discussed in the manuscript. Furthermore, it provides details on additional experiments conducted that were relevant but not central to our studies. The results section in the supplementary file includes figures and tables that are referenced in the manuscript, which are relevant but were not the primary focus of the research. Specifically, the methods used to examine the loss of social interest in the bile duct ligation mouse model of liver disease and the results following A4+ administration are presented (Supplemental methods section 1 and Supplemental results Figure 1). Effects of A4+ and its constituents on zymosan-induced leukotriene B4 production in the rat air-pouch model are also presented (Supplemental results Figure 1). As flow cytometry was used to study the effect of A4+ plant extract on natural killer cell activity, an example of fluorescence-activated cell sorting (FACS) image of a negative control sample is included in Supplemental Figure 4. The effect of A4+ plant extract on natural killer cell activity is presented in Supplemental Table 1. [file 5486728.f1.pdf]

Supplemental Methods

Section 1. Evaluation of the hepato-protective and behavioural effects of the herbal compound A4+ in liver injury models

1a. Bile duct ligation model

Male C57BL/6 mice with initial body weight 22-24 g were used for this study. Animals were housed in an environmentally controlled room with a 12-12-h light-dark cycle and allowed access to food and water ad libitum. The mice were randomly divided into two groups: bile duct resection (BDR) surgery only (control) and BDR plus A4+ (160 mg/ kg/ day administered by oral gavage). Briefly, after 4 days of adaptation, surgery was performed under isoflurane inhalation anesthesia, the common bile duct was exposed through a midline abdominal incision, doubly ligated with 5-0 silk, and sectioned between the ligatures. The incision was then closed by 5-0 silk sutures. Immediately after surgery, mice were treated with antibiotic ointment to the abdominal suture site to prevent infection. Behaviour studies were conducted one day before surgery and 5 and 9 days after the operation.

Sickness behavior was evaluated using two well established methods:

(i) A social investigation paradigm was used to examine loss of social interest (D'Mello et al., 2009). Prior to observation, test and control mice were housed in individual cages for a period of 24 hours. Experiments were performed between 7:00 and 10:00 am on the day prior to surgery and then repeated on days 5 and 9 post-surgery. A 3-4 week old juvenile male C57BL/6 mouse was introduced into the home cage of the test mouse, and behavior was assessed for a period of 10 minutes by two blinded observers. A number of facets of social exploratory behavior were examined, including the total time of social investigation, total time the test mouse remained immobile, and the number of social interactions between the test mouse and a juvenile mouse.

(ii) Open field locomotor activity measurement was used to assess overall mobility (Burak et al., 2002). Prior to observation, test mice were housed in individual cages for a period of 24 hours. Experiments were performed between 7:00 and 10:00 am on day 9 post-surgery. Locomotor activity was measured using an Optivarimax apparatus (Columbus Instruments, Columbus, OH) which records the ambulatory and horizontal movements of a mouse placed in an open field, as measured by the number of times infrared photoelectric beams are broken by the mouse during the observation period (5 min).

1b. Concanavalin A (Con A)-induced hepatitis

The second liver injury model was Concanavalin A (Con A)-induced hepatitis. Concanavalin (Con) A-induced hepatitis is a well-characterized and widely used model of T cell-mediated hepatitis mimicking many aspects of human T cell-mediated liver disease, including autoimmune hepatitis and viral hepatitis (Ajuebor et al., 2005, Ajuebor et al., 2007). For this study, one group of male C57BL/6 mice were pre-treated with saline vehicle (control) and another group with A4+ (640 mg/kg) via oral gavage. The groups received their respective treatments 3 days, 2 days, 1 day and one hour before being given Con A (13.5 mg/kg) intravenously. Twelve hours after injection, mice were killed, and blood was collected for measurement of ALT. Biochemical measurements were performed by Calgary Laboratory Services, Calgary, AB. In addition, livers were dissected and cells processed for flow cytometry analysis (FACS) after staining of different cell surface markers to identify immune cell subsets and to identify cytokine production profiles of these cells using intracellular staining.

Section 2. Evaluation of the anti-oxidant, anti-inflammatory and mucosal protective actions of A4+ and its constituents

2a. Anti-oxidant activity

The anti-oxidant activity of A4+ and its constituents was evaluated using an *in vitro* assay in which a stable free radical (1,1-diphenyl-2-picrylhydrazyl; DPPH) was allowed to interact with the test substance (Vaananen et al., 1992). In the presence of free radical scavengers (i.e., anti-oxidants), this purple-coloured substance, which strongly absorbs at 540 nm, is converted to a colourless compound, 1,1-diphenyl-2-pierylhydra zine. This was detectable as a decrease in absorbance. A 20 µL aliquot of each compound was then added to 180 µL of DPPH (in 95% ethanol; final concentration 100 µM) in a 96-well plate. The compounds tested were Cordia, Annona, and Curcuma extracts. Changes in absorbance at 540 nm were recorded every minute over a 10-min period using a plate scanner.

2b. Anti-inflammatory activity

The anti-inflammatory action of A4+ was evaluated using a widely used in vivo "air pouch" model (Edwards et al., 1981). This model determines the effect of a drug on many different aspects of the inflammatory process. A subdermal 'pouch' was created on the back of the rat by repeated injection of air (Chavez-Pina et al. 2007). The lining of the air pouch, after one week, is histologically similar to the synovial lining, and therefore this model has been used extensively for the characterization of drugs developed for the treatment of arthritis Edwards et al., 1981). After the pouch had been created, an inflammatory event was triggered by injecting zymosan (1 ml of a 1% solution). The test compounds were administered either directly into the air pouch or orally 2 hours prior to injection of zymosan. The rats (n= 5 or 6) were euthanized 4 h after injection of zymosan and the exudates were withdrawn from the air pouch for analysis. The volume of the exudates were measured and aliquots were frozen for

subsequent measurement of concentrations of inflammatory mediators by ELISA (Edwards et al., 1981). The number of leukocytes in the exudates was determined using a Sysmex KX-21N Hematology Analyszer. In addition to a negative control (vehicle), indomethacin was included as a positive control in these experiments. Indomethacin is an anti-inflammatory drug used for the treatment of arthritis, ankylosing spondylitis and gout that has been extensively studied in this model (Chavez-Pina et al. 2007).

2c. Mucosal protective activity

For details for methods see Section 2c of the manuscript.

Section 3. Anti-viral effects of A4+ plant extracts on HCV and HCB infected cells

3a. Hepatitis C studies

The purpose of the first part of this study was to determine the anti-viral effect of A4+ and its constituent herbs on Hepatitis C virus (HCV) infected cells. The studies were carried out using Huh7.5 cells and a tissue-culture adapted strain of HCV, JFH. Cells were seeded, allowed to adhere and establish, followed by 4hr infection incubation with the HCV strain. After infection, cells were washed with fresh media followed by treatment with A4+. Cells were exposed to diluted A4+ powder (0.1, 0.5, 1, 5 and 10 µg/mL in 45% ethanol) for 4 days. Two control groups were established: Ethanol and Negative. Ethanol without A4+ diluted in it was used for the ethanol group and only fresh cell medium was used for the negative control group. This was followed by collection of supernatant and cells for viral titering. The studies were repeated three times and in each, results showed that the A4+ powder, and in particular the A4+L component of the formulation, had significant antiviral activity. Intracellular and extracellular fractions were measured after 4 days of herbal treatment.

To further assess the antiviral effects of A4+L, viral protein levels were visualized by Western blot. Cells were plated, infected, and herbal treatment conducted as previously described. After treatment, cells were lysed with RIPA buffer to release cell contents and prevent protein degradation. Protein levels were quantified with a BioRad Protein assay, to ensure that the same amount of protein was added to each well. Two HCV antibodies were used to determine viral quantity: NS3 and core.

3b. Hepatitis B studies

The HBV antiviral studies used both HepG2.2.15 and HepAD38 cells, which express HBV constitutively under the presence of the specific promoters. Cells were seeded on 6 well plates at 1 x 10^6 cells/well, and were left to adhere overnight before any herbal treatments were added in specified concentrations. Cells were treated for 4 days, at which time supernatant and cells were harvested for HBV quantification. Non-viral nucleic acid was digested with DNase I and RNase followed by viral precipitation with PEG 8000. Viral envelope was digested with proteinase K and SDS overnight and nucleic acid was extracted by phenol-chloroform. DNA was precipitated by ethanol precipitation and re-suspended in 10uL of water.

3c. Viability Assays

Cell viability during herbal treatment was determined using an MTT assay. Cells were seeded at approximately 20% confluency in a 96 well plate. After overnight incubation, whereby cells became adherent, a 4 hour infection cycle then occurred. This was followed by a 24 hour herbal treatment. Cells were washed with PBS and fresh media was added, which contained 0.5mg/mL MTT. Cells were then cultured for 16 hours. Media was aspirated off and resulting substrate was dissolved in 100uL 0.1N HCl/isopropanol. Absorbance was read at 570nm. As the number of cells increase, the amount of MTT metabolized also increases,

resulting in a larger quantity of purple formazan formation, and therefore the development of a higher absorbance.

3d. Effect of Short-term exposure to A4+ plant extract on Natural Killer Cell Activity

Herbs received as powders were dissolved in 45% ethanol, and all herbs were diluted in 20% sucrose. C57B/6 mice were given 100 uL of sucrose, ethanol, or herb (1.3 mg/day) by gavage and animals were treated over 14 days. Mice were then euthanized, and their spleens were collected. A cell suspension was made in isolation media by passing the spleen through a fine mesh sieve. Cells were washed 2 times with fresh isolation media, and red blood cells were lysed with 1 wash in RBC lysis buffer. NK cells were then purified using an Easy Sep Mouse NK Cell Enrichment Kit. Purified NK cells were incubated with YAC-1 cells at a 1:5 ratio at 37˚C for 4 hours in the presence of a CD107a antibody (degranulation marker). The last 15 minutes of the incubation included antibodies CD3, CD45, NK1.1 and DX5. Cells were then fixed with 1% paraformaldehyde, permeabilized in 0.1% saponin, and re-suspended in FACS buffer. Purified NK cells were analyzed by FACS.

Supplemental Results

Section 1. Evaluation of the hepato-protective and behavioural effects of the herbal compound A4+ in liver injury models

1a. Social investigation behavior

The times spent in social investigation behavior of the control and A4+ treated BDR mice were not significantly different at days 5 and 9 post-surgery (Supplemental Figure 1). Baseline activity was assessed just prior to surgery on the same day. Although the number of social interactions appeared to be greater for the A4+ treated mice compared to controls at

days 5 and 9, the differences were not significantly different. The observed enhancement of interactions may be a result of the variability between animals.

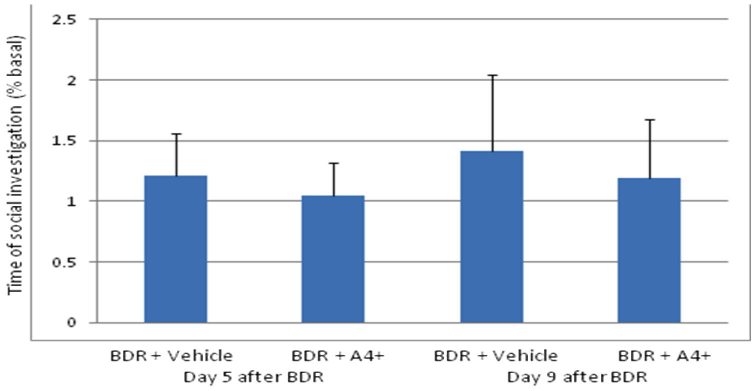

Supplemental Figure 1. Time that the test BDR mouse spent in social investigation behaviour with the juvenile mouse (as % of basal activity as determined on day before surgery) at days 5 and 9 post-BDR surgery. Two groups of BDR mice were studied: BDR + vehicle and BDR + A4+ (160 mg/kg/day by oral gavage). Bars represent the means $\pm$ SD of data from 7-8 mice/group. BDR vs BDR + A4+ groups are not significantly different at day 5 or day 9.

Section 2. Evaluation of the anti-oxidant, anti-inflammatory and mucosal protective actions of A4+ and its constituents

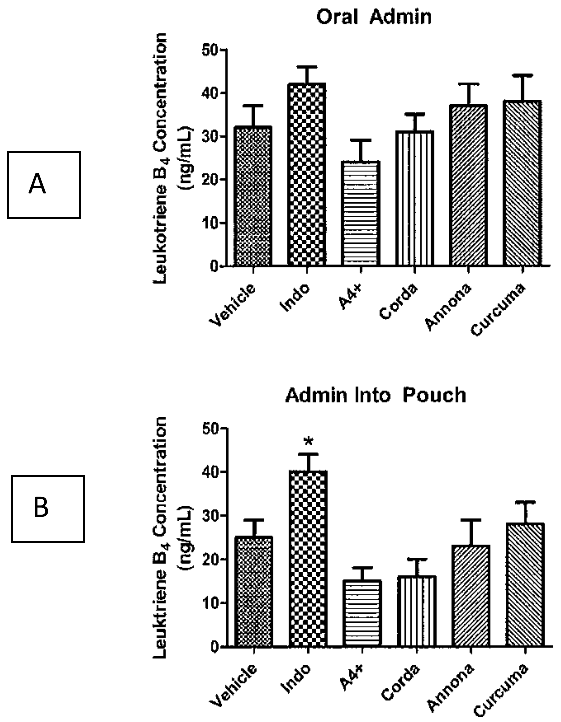

Supplemental Figure 2. Effects of A4+ and its constituents on zymosan-induced leukotriene B_4 production in the rat air-pouch model. **A:** When A4+ was given orally 2 hours prior to the injection of zymosan into the air-pouch, A4+ and its constituents had no significant effect. Similarly, indomethacin (1 mg/kg) had no significant effect. A4+ was administered at 100 mg/kg, while Cordia, Annona and Curcuma were administered at doses of 80, 10 and 10 mg/kg, respectively. **B:** Administration of A4+ or its constituents directly into the air-pouch 2 hours prior to zymosan administration had no effect on leukotriene B_4 levels. A4+ was administered at 10 mg/kg, while Cordia, Annona and Curcuma were administered at 8, 1 and 1 mg/kg, respectively. In contrast, indomethacin produced a significant increase in leukotriene B_4 levels. Results are shown as mean± SEM (n= 5-6 rats/group). *$p<0.05$, versus the vehicle-treated group (ANOVA and Dunnett's Multiple Comparison test).

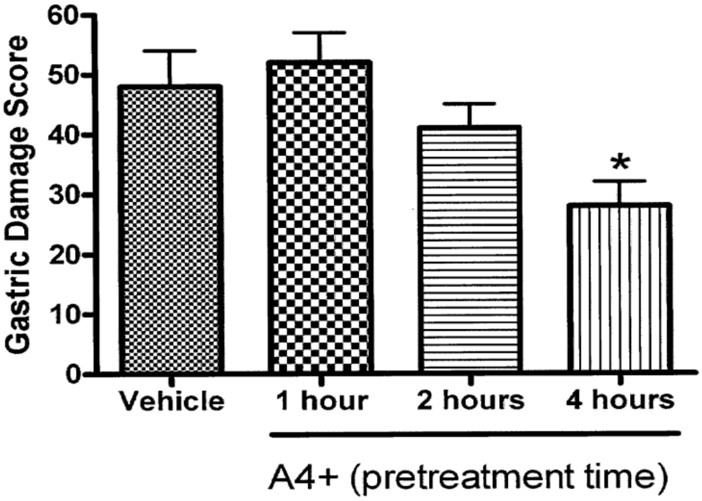

Supplemental Figure 3. Time-dependent effects of A4+ on indomethacin-induced gastric damage in rats. Groups of 5 rats each received A4+ (300 mg/kg orally) 1, 2 or 4 hours prior to oral administration of indomethacin (20 mg/kg). *$p<0.05$ versus the vehicle-treated group.

Section 3. Anti-viral effects of A4+ plant extracts on HCV and HCB infected cells

Effect of Short-term exposure to A4+ plant extract on Natural Killer Cell Activity

NK cells are a small population of lymphocytes that have an ability to target specific cells and induce cell death. Fluorescently labelled antibodies bind to CD107a, the protein found on the inner surface of the granular membrane, which becomes exposed on the cell surface during degranulation. In order to distinguish the NK population, antibodies DX5, NK1.1, CD3 and CD45, were used to label cells. As cells pass through the FACS machine, the various labels are detected, allowing for cell type determination. Cells positive for both NK1.1 and CD107 were the NK cells of interest. An example of FACS image of a negative control sample is shown in Supplemental Figure 4.

The NK assays were split into two groups, with the controls being done for each group. In both cases, NK activity is approximately 12% higher after ethanol treatment, however this data may be skewed due to one outlier in the ethanol treatment during the second round (this mouse may have had a secondary infection after ethanol gavage and possibly getting ethanol in the lungs). When this data point was removed, the difference between the negative control and the ethanol treatment was smaller. Treatment with A4+ and A4+L did not differ from the controls (Supplemental Table 1). Statistical analysis was calculated for results shown on Supplemental Table 1, however no treatment was determined to produce a significant enhancement or decrease in NK activity.

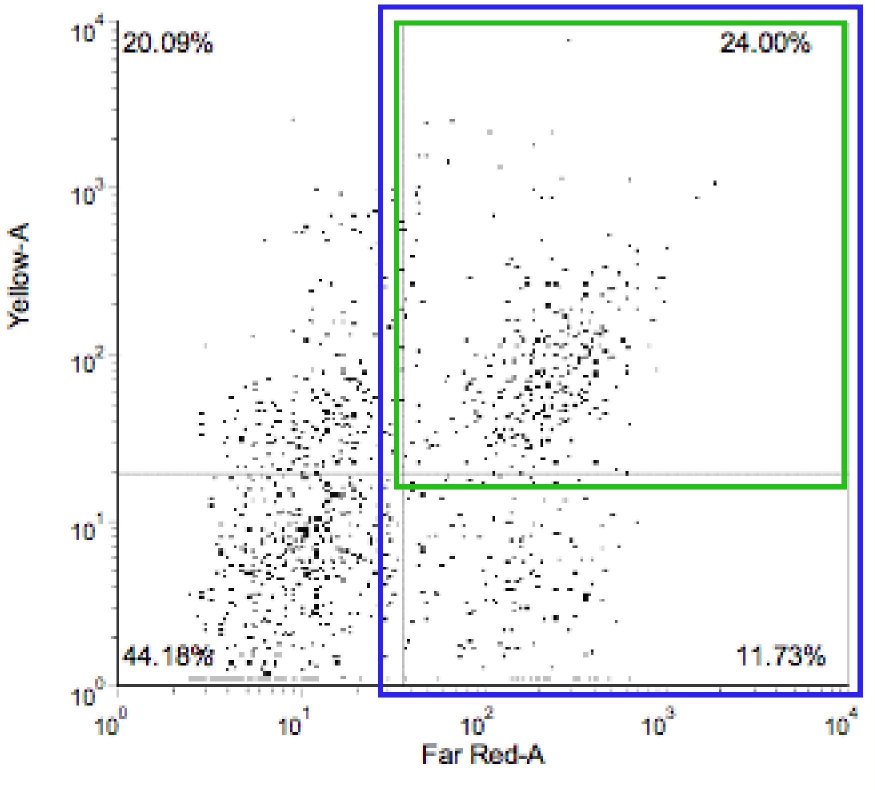

Supplemental Figure 4. A FACS image of the negative control sample. Each dot represents a cell that has passed through the detector. The x-axis is the fluorescent marker for NK cells, where the y-axis is the fluorescent marker for degranulation (CD107a). The cells within the blue box are NK positive cells, and the green box within the blue box are NK cells that have degranulated. The percentage of positive cell was calculated by dividing the NK+CD107a+ positive cells by the total NK population.

Treatment	Repeat 1	Repeat 2	Repeat 3	Average
Negative	54.2	56.9	56.0	55.7
Neg-Ethanol	67.2	85.2	51.7	68.0
A4+	61.9	54.8	63.0	59.9
A4+L	64.6	15.8	38.8	39.7

Supplemental Table 1. Second round of NK assays, in triplicate, showing the percentage of NK positive cells that have undergone degranulation. These treatments did not produce a significantly different effect.

References

Ajuebor MN, Wondimu Z, Hogaboam CM, Le T, Proudfoot AE, Swain MG. CCR5 deficiency drives enhanced natural killer cell trafficking to and activation within the liver in murine T cell-mediated hepatitis. Am J Pathol. 2007;170(6):1975-88.

Ajuebor MN, Aspinall AI, Zhou F, Le T, Yang Y, Urbanski SJ, et al. Lack of chemokine receptor CCR5 promotes murine fulminant liver failure by preventing the apoptosis of activated CD1d-restricted NKT cells. J Immunol. 2005;174(12):8027-37.

Burak KW, Le T, Swain MG. Increased sensitivity to the locomotor-activating effects of corticotropin-releasing hormone in cholestatic rats. Gastroenterology. 2002;122(3):681-8.

Chavez-Pina AE, McKnight W, Dicay M, Castaneda-Hernandez G, Wallace JL. Mechanisms underlying the anti-inflammatory activity and gastric safety of acemetacin. Br J Pharmacol. 2007;152(6):930-8.

D'Mello C, Le T, Swain MG. Cerebral microglia recruit monocytes into the brain in response to tumor necrosis factoralpha signaling during peripheral organ inflammation. J Neurosci. 2009;29(7):2089-102.

Edwards JC, Sedgwick AD, Willoughby DA. The formation of a structure with the features of synovial lining by subcutaneous injection of air: an in vivo tissue culture system. J Pathol. 1981;134(2):147-56.

Vaananen PM, Keenan CM, Grisham MB, & Wallace JL (1992). Pharmacological investigation of the role of leukotrienes in the pathogenesis of experimental NSAID gastropathy. Inflammation 16: 227-240.
